# All aspects of sciatic nerve injection injury: an experiment with 78 rats

**DOI:** 10.55730/1300-0144.5499

**Published:** 2022-08-04

**Authors:** Mete ZEYNAL, Hakan Hadi KADIOĞLU

**Affiliations:** Department of Neurosurgery, Faculty of Medicine, Atatürk University, Erzurum, Turkey

**Keywords:** Sciatic nerve, injection injury, peripheral nerve, electroneuromyography

## Abstract

**Background/aim:**

In this study, we evaluate sciatic nerve injuries due to intramuscular injections, which is an important medicolegal problem frequently encountered in medical practice, with an extended experimental rat model of peripheral nerve injury.

**Materials and methods:**

A total of 78 male Wistar albino rats were divided into five main groups, including a control group, a sham saline group, and groups that received benzathine penicillin G, diclofenac sodium, and dexamethasone, respectively. These pharmaceutical agents were applied to the sciatic nerves of all rats after exploration in the epineurial, endoneurial, and intrafascicular compartments, excluding the control group. Outcomes were evaluated for all rats and their sciatic nerves according to functional, electrophysiological, and histopathological results.

**Results:**

Injuries were most evident in the groups that received penicillin G and diclofenac sodium, and this finding was statistically significant. It was also found that endoneurial and intrafascicular injections may cause more harm than epineurial injections.

**Conclusion:**

We have demonstrated that any medical injections applied to the epineurial, endoneurial, or intrafascicular compartments of the sciatic nerve may cause functional and electrophysiological loss with or without deterioration of the peripheral nerve architecture.

## 1. Introduction

Intramuscular injections (IMIs) are common medical applications today as they can be easily applied to the gluteal, quadriceps, and deltoid muscles. However, as a result of incorrect applications, severe disorders may arise. According to global data from the World Health Organization, 50% of all IMIs are administered incorrectly and 75% are administered unnecessarily [1,2]. Abscesses, necrosis, contracture, periostitis, and peripheral nerve injuries may occur following incorrect applications of IMIs [1–5]. Peripheral nerve injection injuries are caused by the application of the needle directly to the nerve fiber or somewhere near the nerve fiber and they are strongly associated with the neurotoxic effects of the drug; the penetration of the needle is not the reason for the nerve injury [4]. High volumes of medications may also cause nerve injury due to compression. Experimental studies have shown that minimal damage may occur after epineurial injections of pharmaceutical agents, but for severe damage, endoneurial or intrafascicular penetration of the agent is necessary [2].

The sciatic nerve is the nerve that is most commonly injured by IMIs. Peripheral nerve injection injury is reported at a rate of 2% in the literature [5–7]. Sciatic nerve injury due to gluteal IMI has a wide range of clinical presentations, but the most common complaints are posterolateral gluteal neuropathic pain and sensorial or motor deficiencies [8,9]. The clinical symptoms of the patient may be observed immediately after the injection or may develop later [7].

There are many anatomical, clinical, and experimental studies in the literature regarding nerve injection injuries, but the present study is unique as it was designed to compare all possible injection injury mechanisms. In this experimental study, we evaluate the effects of the most commonly used IMI drugs (benzathine penicillin G, diclofenac sodium, dexamethasone, and saline) when injected into the epineurial, endoneurial, and intrafascicular compartments of the peripheral nerve in terms of electromyographic, functional, and histopathological characteristics.

## 2. Materials and methods

This experimental study has been done under the approval of XXX University, Animal Experiments Local Ethics Committee Presidency with reference number 75296309-050.01.04-E.1600181052/132.

This study was performed with 78 male Wistar albino rats that weighed 200–250 g. The rats were divided into five main groups, including a control group, a sham saline group, and groups that received benzathine penicillin G, diclofenac sodium, and dexamethasone, respectively. Excluding the control group, all groups were also divided according to the epineurial, endoneurial, and intrafascicular drug application compartments. Each subgroup consisted of 6 rats. Six rats were also preserved to reveal normal histology. During this study 11 rats died and they were excluded out from the result and statistical analysis.

**Figure 1:**
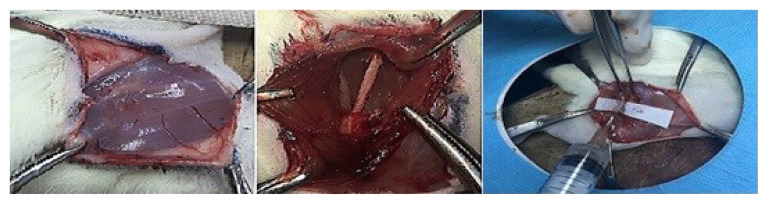
Surgical technique: dissection of the gluteal region of the rat, exposure of the sciatic nerve, and endoneurial injection.

After 12 h of fasting, the rats were prepared for the experiment. Ketamine hydrochloride and xylazine were applied intraperitoneally for general anesthesia. A right gluteal oblique incision was performed and after muscular dissection, the sciatic nerves of the rats were exposed. Initial electromyography (EMG) recordings were performed for the sciatic nerve and the results were recorded. For these EMG recordings, a Cadwell Cascade Elite electro-neuromonitoring device was used (Cadwell, Kennewick, WA, USA). The pharmaceutical agents were injected with a 30-G needle into the appropriate compartment for each subgroup in an amount of 0.5 cc. (Figure 1). Acute amplitude changes were recorded by EMG after the injections as the second EMG recording. The injection sites were marked with 3/0 silk muscle suturing to make it easier to identify the injury zone in the follow-up period. The wounds were closed properly.

In the postoperative period, rats were placed in single cages in a room with normal temperature and daily dressings of povidone-iodine were applied. Amoxicillin-clavulanic acid was added to animal feed as a prophylactic antibiotic. After 14 days, the third EMG recordings were performed for all rats and injured sciatic nerves were excised. During electrophysiological examinations, motor action potentials were recorded in millivolts (mV) and analyzed as needle electrode readings. The first amplitude value recorded from the sciatic nerve after the initial exploration before the application of any pharmaceutical agents was taken as the first reading. The second amplitude values recorded after the application of pharmaceutical agents to the epineurial, endoneurial, and intrafascicular compartments were taken as the second readings. The third amplitude values recorded during the reexploration of the sciatic nerve in the final stage of the experiment after 14 days were taken as the third reading (Table 1). During the 14-day period following the application of the pharmaceutical agents, neurological evaluations were also performed based on walking track analysis, extensor postural thrust (EPT), and the presence of drop-foot (Tables 2, 3) [10,11].

Sciatic nerve samples were collected for pathological examination to reveal the sciatic nerve morphology and presence of inflammation, edema, and congestion by H&E staining. Masson’s trichrome stain was applied to reveal perineural fibrosis and toluidine blue stain was applied to reveal Wallerian degeneration. All of these samples were examined by two independent pathologists under a light microscope (Axio Scope A1, ZEISS, Oberkochen, Germany). Histopathological data were evaluated with a modified histopathological grading scale based on the criteria of Faroni et al. (Table 4) [12].

### 2.1. Statistical analysis

All statistical analyses were done with IBM SPSS Statistics 19.0 for Windows (IBM Corp., Armonk, NY, USA). The normal distribution of the variables was analyzed by Shapiro-Wilk. Normal distribution was not detected in most of the variables, so the Kruskal Wallis test was used for intergroup comparison and the Friedman test for in-group repeated measurement comparison.

## 3. Results

Rats that received epineurially injected saline, penicillin G, diclofenac sodium, and dexamethasone did not show any differences in EPT tests and no drop-foot or dragging of the feet was observed in these rats. In the epineurial injection subgroups, electrophysiological changes were not recorded. Macroscopically, there was only mild congestion, and in microscopic evaluations, only Grade 1 changes were observed.

**Figure 2:**
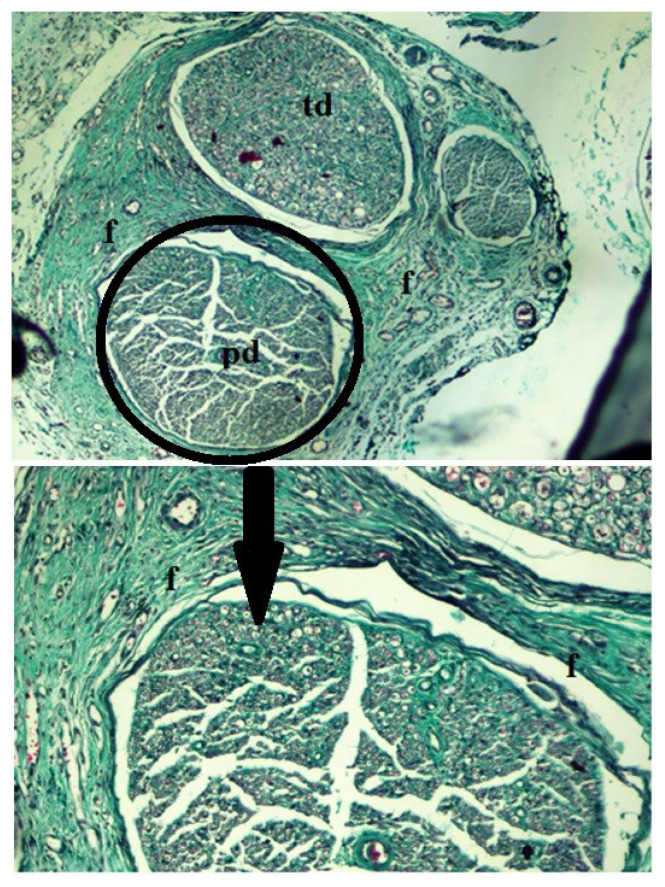
Fibrosis after endoneurial penicillin G injection: peroneal branch (pd) and tibial branch (td) are surrounded by fibrosis with fibroblast proliferation (f) (Masson’s trichome staining, 40× magnification).

Endoneurial injections revealed no significant differences, except for penicillin G and diclofenac sodium. We found statistically significant differences in the EPT test results for the subgroup endoneurially injected with penicillin G and diclofenac sodium (p < 0.05). In penicillin G subgroup both drop-foot and dragging of the feet were observed in these rats (p < 0.05). Electrophysiological changes also had statistical importance in all endoneurial injection groups (p < 0.05). Furthermore, histopathologically, perineural fibrosis was evaluated as Grade 3 in this subgroup and intraneural inflammatory cell infiltration and Wallerian degeneration were evaluated as nearly Grade 2 (Figure 2).

**Figure 3:**
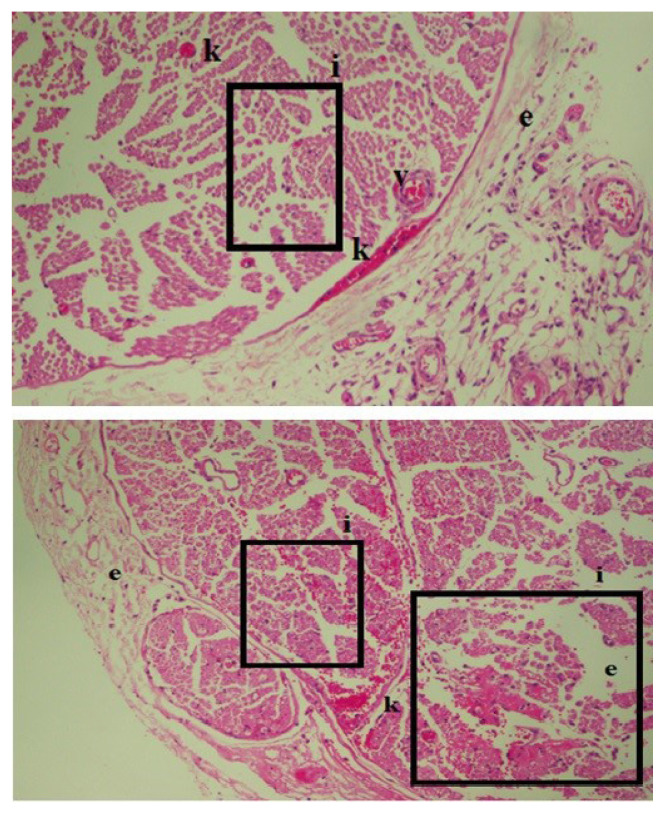
Inflammation and edema after interfascicular injection of penicillin G: intraneural inflammatory cell infiltration (i), edema due to inflammatory response (e), and congestion are seen (H&E staining, 40× magnification).

**Figure 4:**
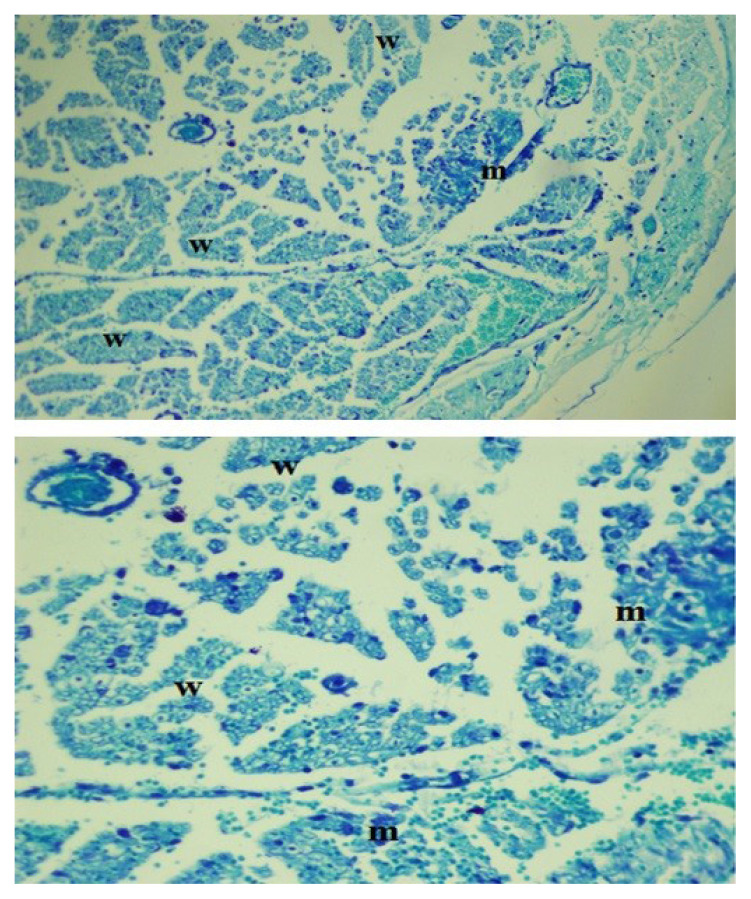
Wallerian degeneration after intrafascicular penicillin G injection: axonal injury and myelin loss (m) with Wallerian degeneration (w) (toluidine blue staining, 40× magnification).

Statistically significant results were also found among the intrafascicular injection subgroups. The EPT test results showed an average difference of 13.2 g for the penicillin G subgroup and an average difference of 6.4 g for the diclofenac sodium subgroup (p < 0.05). Interestingly in dexamethasone subgroups EPT test resulted as statistically significant (p < 0.05). There were also statistically significant differences in all intrafascicular subgroups among the results for electrophysiological changes (p < 0.05). Microscopic histopathological evaluations showed Grade 2–3 inflammatory cell infiltration and Wallerian degeneration for both penicillin G and diclofenac sodium, but no fibrosis was observed (Figures 3 and 4).

Drop-foot developed over the course of 14 days for all rats that received endoneurial and intrafascicular injections of penicillin G and diclofenac sodium, and the results of walking track analysis for foot dragging were positive and statistically significant in these subgroups (p < 0.05). EPT analysis showed no statistically significant results for any pharmaceutical agents administered by epineurial injection (p > 0.05). EPT results obtained after the administration of penicillin G to the intrafascicular compartment were found to be statistically significant (p < 0.05) upon comparisons of all three compartment groups for endoneurial injections. Endoneurial injection of diclofenac sodium also showed statistically significant results (p < 0.05) in EPT analysis. Furthermore, the subgroup that received intrafascicular dexamethasone had statistically significant results (p < 0.05) in the third EPT analysis when compared to the epineurial and endoneurial injections of the considered pharmaceutical agents.

The presence of drop-foot and positive results in walking track analysis were statistically correlated with Wallerian degeneration and the presence of perineural fibrosis, which are highly associated with neural degeneration and clinical disorders. Both of these findings were present for the subgroup endoneurially injected with penicillin G and the subgroup intrafascicularly injected with diclofenac sodium.

The results for all three EMG readings were statistically examined and correlations with functional evaluations were sought. There were no statistically significant differences in this regard after the epineurial drug injections (p > 0.05). Endoneurial injections of all drugs had a statistically significant relationship with decreased EMG amplitudes (p < 0.05). Decreased amplitudes in the subgroups administered intrafascicular injections of penicillin G and diclofenac sodium were also statistically significant (p < 0.05). When all of the considered compartments and pharmaceutical agents were compared, the most statistically significant low amplitudes were seen in the penicillin G and diclofenac sodium subgroups (p < 0.05).

## 4. Discussion

In neurosurgical practice, peripheral nerve injuries are often seen. The most common causes of these injuries are trauma, crushing, exposure to pressure, laceration, surgical procedures around the hip joint, stretching, and chemical injuries. Injection neuropathies are categorized as chemical injuries and the annual incidence is reported as 2% [6,7]. The sciatic nerve (55%), radial nerve (24%), and femoral nerve (15%) are the nerves for which injection neuropathies are most commonly observed [5]. Naturally, there are vascular structures and nerves inside or around muscle groups, and the inattentive administration of injections in these important structures may cause injuries. There is serious potential for morbidity as a result of the neurotoxic effects of pharmaceutical agents in the event of such injections into peripheral nerves [1,2,8,13,14].

In a study conducted by Kline et al., the etiology of 136 cases of 230 sciatic nerve injuries was reported to be gluteal intramuscular injection [3]. The exact incidence rate of such injuries may be higher than what is reported because intramuscular injections and injection injuries may not always be officially recorded in efforts to protect health professionals from medicolegal troubles or for other sociocultural reasons. Thus, the true incidence of these injection injuries may be masked.

The ventrogluteal region and the vastus lateralis of the quadriceps femoris muscles are often preferred for IMIs for easy applicability, adequate distance from important anatomic structures, and the capability of receiving larger drug volumes. Ventrogluteal region injections are also advantageous because it is easy to determine the injection site, making IMIs safer, and the injection site does not change due to positioning, so IMIs can be administered here regardless of whether the patient is supine, prone, or laterally positioned [1]. IMIs should be applied more carefully for obese patients, because finding the right injection site may be a challenge due to palpation difficulties. The gluteal muscles are not yet fully developed in pediatric patients and gluteal IMIs should therefore not be applied in this population. They should be avoided for cachectic patients for a similar reason, as was confirmed by Yaremeyeva et al. in a study of injury predisposition among cachectic patients [14]. It should be noted that the sciatic nerve has seven different variations, but all variations associated with the dorsogluteal region should be avoided while administering IMIs. On the other hand, the ventrogluteal region is safe if proper IMI applications are administered according to this anatomical knowledge [1,15–17]. This accordingly raises questions of medicolegal issues or malpractice.

The biochemical characteristics of an administered drug are also important factors for the occurrence of neuropathy. The drugs commonly administered by IMI are produced in solution, suspension, or emulsion forms. Fat-soluble vitamins, ferrous preparations, and procaine penicillin accumulate in muscle tissues and are absorbed slowly into the systemic circulation [17]. These kinds of pharmaceutical agents may leak out of the tissues in which they have accumulated and reach neural structures, causing neural injuries to occur [18]. The amount of the injected drug is another important issue for injection neuropathy because higher volumes are absorbed more slowly, making leakage and pressure more possible. Drug injection volumes are recommended as 3–5 mL for the gluteal muscles and 2 mL for the deltoid muscles [1].

In cases of injection neuropathy, the damage specifically occurs around the nerve, in the neural sheath, or in the neural fibers. The experiment presented here was designed with that fact in mind. The chosen pharmaceutical agents (saline, penicillin G, diclofenac sodium, and dexamethasone) are commonly used in general medical practice and may have neurotoxic effects. The injection of these agents into the epineurial, endoneurial, and intrafascicular compartments has been evaluated in this study neurologically, electrophysiologically, and histopathologically. With this experimental design, we evaluated the effects of each pharmaceutical agent for each of the three compartments. Any pharmaceutical agent applied to a peripheral nerve, regardless of its chemical properties, can cause an inflammatory reaction in the epineurial, endoneurial, or intrafascicular compartments; thus, edema and fibrosis may occur. This inflammatory response may cause nerve injury in turn as a result of microcirculatory changes and ischemia [7,8,19].

The epineurial compartment is important because it is the space in which pathological changes occur with the compression of the peripheral nerve due to fibrosis or scar formation caused by inflammatory changes [2,8,13,10,20]. In this study, we demonstrated that the needle penetration into the nerve body alone is not what causes the injury. The endoneurial compartment works as a shock absorber for peripheral nerves and it can stretch longitudinally. The injection of a drug into this compartment causes nerve injury by increasing both the pressure and chemical toxicity. This increased pressure in the endoneurial compartment results in ischemia and damage in sensitive neural tissues by disturbing the microvascular circulation. It is also important to consider whether the basal membrane has been destroyed in the fascicule or not. After IMIs, intramuscular fibroblast proliferation occurs, causing fibrosis, and the fibrosis subsequently results in constriction of the tissues surrounding the peripheral nerve. By this mechanism, nerve perfusion is decreased [21,20]. Nerve fibers are directly exposed to neurotoxic agents when injections are applied to the endoneurial compartment. On the other hand, extrafascicular injections do not cause neural damage every time, but injuries may occur if the injected agents are highly neurotoxic, such as penicillin G, diazepam, or chlorpromazine. Our study and other previous animal experiments have shown that even saline, which has no neurotoxic effects if injected into the intrafascicular compartment, may cause edema in such cases. This causes increased intrafascicular pressure and ischemia. More neurotoxic agents may cause axonal destruction and Wallerian degeneration in addition to edema [7,8,22]. In our study, similar pathological changes were observed in the subgroups that received penicillin G and diclofenac sodium.

Neurological examinations and electrophysiological studies yielded parallel results. The histopathological changes in the penicillin G subgroups were the most statistically significant; thus, the neurotoxicity of the penicillin drug group is very high [3,2,17]. This was followed by the diclofenac sodium and saline subgroups in the present study. Intraneural inflammatory cell infiltration was found to be significant for the penicillin G and diclofenac sodium groups. Dexamethasone was the mildest agent of this study in terms of histopathological changes. This is probably a result of the antiinflammatory effects of dexamethasone. In contrast, it was interesting that endoneurial saline infusion caused Wallerian degeneration in this study. This may have been the result of increased pressure and ischemia arising from fast injections performed manually [2,15]. Low-pressure injections may cause transient changes, but fast high-pressure injections may cause severe functional and structural changes [14,17]. Hadzic et al. evaluated neurological results and changes with increasing pressure in dogs as a result of intrafascicular injections and demonstrated that high pressure may cause severe neurological disorders and fascicular damage [17]. Kokhan et al. conducted a study with 22 patients and showed that local anesthetic injections of 1 mL may raise the pressure in the sciatic nerve by 40 mmHg [23]. Therefore, the amount of the drug injected is also important because pressure increases with that amount, as does neural damage.

In conclusion, our study has demonstrated that any IMI applied to the region of the sciatic nerve may cause histopathological damage depending on the chemical and neurotoxic properties of the injection. Both permanent and temporary neurodeficiencies may occur according to the injection compartment that is used. Furthermore, the amount, injection speed, and neurotoxicity of the pharmaceutical agent will also affect the clinical presentation. According to our study and the relevant literature, permanent or temporary sciatic nerve injection injuries may occur if injections are applied to the piriform fossa with or without use of the endoneurial compartment. Following appropriate application procedures will protect the sciatic nerve even in the event of nerve variations.

## Figures and Tables

**Table 1:** Comparison of all groups according to EMG recordings. mV: milivolt, p^*^: Friedman Test, p^**^: Kruskal Wallis Test

Test	Epineurial 1st EMG (mV)	Epineurial 2nd EMG (mV)	Epineurial 3rd EMG (mV)	p^*^	Endoneurial 1st EMG (mV)	Endoneurial 2nd EMG (mV)	Endoneurial 3rd EMG (mV)	p^*^	Intrafascicular 1st EMG (mV)	Intrafascicular 2nd EMG (mV)	Intrafascicular 3rd EMG (mV)	p^*^
Drug												
**Sham (Saline)**	756.20 ± 90.39 721 (649–878) (n = 5)	654.20 ± 104.07 611 (552–783) (n = 5)	640.60 ± 93.79 600 (529–741) (n = 5)	**0.015** (post hoc: 3rd -1st)	579.20 ± 144.36 616 (362–748) (n = 5)	497.20 ± 197.43 600 (280–700) (n = 5)	496.60 ± 187.92 597 (279–690) (n = 5)	**0.015** (post hoc: 3rd -1st)	547 ± 237.62 556.50 (200–864) (n = 6)	474.50 ± 200.21 522 (176–716) (n = 6)	454.33 ± 193.99 506 (160–690) (n = 6)	**0.002** (post hoc: 3rd -1st)
**Penicillin G**	534.17± 163.95 558 (276700) (n = 6)	518.17 ± 163.54 548.50 (254–690) (n = 6)	506.33 ± 164.27 534 (246–681) (n = 6)	**0.002** (post hoc: 3rd -1st)	708 ± 36.42 711 (661–749) (n = 4)	395.50 ± 88.79 380 (307–515) (n=4)	329 ± 66.67 315.50 (271–414) (n = 4)	**0.018** (post hoc: 3rd -1st)	730.40 ± 129.66 701 (605–950) (n = 5)	570.60 ± 160.83 501 (419–835) (n = 5)	363.20 ± 144.93 294 (254–600) (n = 5)	**0.007** (post hoc: 3rd -1st)
**Diclofenac sodium**	523.20 ± 105.81 597 (399–603) (n = 5)	500.40 ± 112.49 554 (360–599) (n = 5)	505.60 ± 142.20 513 (355–685) (n = 5)	0.091	678.80 ± 201.11 700 (455–986) (n = 5)	557.20 ± 131.28 511 (439–749) (n = 5)	531.20 ± 109.18 489 (430–689) (n = 5)	**0.007** (post hoc: 3rd -1st)	718.60 ± 183.82 737 (497–900) (n = 5)	596.20 ± 185.99 600 (311–818) (n = 5)	519.40 ± 157.68 583 (303–696) (n = 5)	**0.007** (post hoc: 3rd -1st)
**Dexamethasone**	558.20 ± 140.85 619 (336–701) (n = 5)	538.80 ± 140.67 600 (314–675) (n = 5)	512 ± 146.99 595 (310–651) (n = 5)	**0.007** (post hoc: 3rd -1st)	580.20± 270.31 505 (283–991) (n = 5)	515 ± 232.26 455 (254–874) (n = 5)	487.20± 203.76 432 (250–800) (n = 5)	**0.008** (post hoc: 3rd -1st)	531 ± 96.64 524 (387–637) (n = 5)	353.80 ± 84.55 399 (231–436) (n = 5)	341.60 ± 84.99 381 (222–431) (n = 5)	**0.007** (post hoc: 3rd -1st)
**p** ^**^	0.018	0.340	0.386		0.334	0.507	0.205		0.115	0.094	0.190	

**Table 2:** Comparison of all groups in the EPT (extensor postural thrust) test. g: gram, p^*^: Friedman Test, p^**^: Kruskal Wallis Test

Test	Epineurial 1st EPT (g)	Epineurial 2nd EPT (g)	Epineurial 3rd EPT (g)	p^*^	Endoneurial 1st EPT (g)	Endoneurial2nd EPT (g)	Endoneurial3rd EPT (g)	p^*^	Intrafascicular1st EPT (g)	Intrafascicular2nd EPT (g)	Intrafascicular3rd EPT (g)	p^*^
Drug												
**Sham (Saline)**	29.80 ± 2.68 30 (27–34) (n = 5)	29.20± 2.95 30 (25–33) (n = 5)	27.80±1.92 28 (25–30) (n = 5)	0.146	27.40 ± 3.05 27 (24–31) (n = 5)	27 ± 3.39 26 (23–31) (n = 5)	26.40 ± 3.85 26 (21–31) (n = 5)	0.082	27.50 ± 3.15 27.50 (24–33) (n = 6)	26.33 ± 3.39 26.50 (22–32) (n = 6)	25.17 ± 4.07 24.50 (22–33) (n = 6)	**0.018** (posthoc: 3rd -1st)
**Penicillin G**	27.17 ± 3.13 28 (23–31) (n = 6)	26.83± 3.31 27.50 (22–31) (n = 6)	26.67±3.50 27.50 (22–31) (n = 6)	0,097	29.75 ± 2.75 29.50 (27–33) (n = 4)	27 ± 2.45 26.50 (25–30) (n = 4)	25.50 ± 1.29 25.50 (24–27) (n = 4)	**0.022** (post hoc: 3rd -1st)	29.20 ± 3.27 28 (25–33) (n = 5)	18.40 ± 1.34 19 (17–20) (n = 5)	16 ± 1 16 (15–17) (n = 5)	**0.008** (posthoc: 3rd -1st)
**Diclofenac sodium**	27.40 ± 2.70 29 (24–30) (n = 5)	26.80± 2.59 28 (24–29) (n = 5)	26.40±2.30 27 (24-29) (n = 5)	0.060	27.60 ± 3.85 27 (23–32) (n = 5)	26.60 ± 3.29 26 (23–30) (n = 5)	25.80 ± 2.77 26 (23–29) (n = 5)	**0.022** (post hoc: 3rd -1st)	27 ± 2.45 27 (23–29) (n = 5)	22.80 ± 4.21 24 (17–27) (n = 5)	20.60 ± 5.22 19 (16–29) (n = 5)	0.104
**Dexamethasone**	26.80 ± 2.68 28 (23–29) (n = 5)	26.20± 2.59 27 (23–29) (n = 5)	25.80±2.39 26 (23-29) (n = 5)	0.060	26.80 ± 3.90 29 (22–30) (n = 5)	25.80 ± 3.11 27 (22–29) (n = 5)	25 ± 2.35 26 (22–27) (n = 5)	0.060	29.80 ± 2.17 29 (28–33) (n = 5)	26 ± 3.08 25 (22–30) (n = 5)	23.60 ± 4.16 22 (20–29) (n = 5)	**0.008** (posthoc: 3rd -1st)
**p** ^**^	0.418	0.318	0.618		0.571	0.873	0.930		0.305	**0.015**	**0.009**	

**Table 3:** All groups were compared for drop-foot and dragging existance (number of test subjects / positive existance).

Test	Epineurial Drop-foot, dragging	p^*^	Endoneurial Drop-foot, dragging	p^*^	Intrafascicular Drop-foot, dragging	p^*^
Drug						
**Sham (Saline)**	5/0	N/A	5/0	N/A	6/0	N/A
**Penicillin G**	6/0	N/A	4/4	**0.018**	5/5	**0.007**
**Diclofenac sodium**	5/0	N/A	5/0	N/A	5/5	**0.007**
**Dexamethasone**	5/0	N/A	5/0	N/A	5/0	
**p** ^**^	N/A		**<0.001**			**<0.001**

p^*^ Friedman Test, p^**^ Kruskal Wallis Test

**Table 4:** Modified histopathological grading scale from Faroni et al.

Pathology	Score	Criteria (cross-section, 40× magnification)
**Fibrosis**	*Grade 1*	<150 fibroblasts
	*Grade 2*	100–150 fibroblasts
	*Grade 3*	>150 fibroblasts
**Wallerian degeneration**	*Grade 1*	<25% degeneration
	*Grade 2*	25%–75% degeneration
	*Grade 3*	>75% degeneration
**Edema**	*Grade 1*	<25%
	*Grade 2*	25%–75%
	*Grade 3*	>75%
**Inflammatory cell infiltration**	*Grade 1*	0–10 neutrophils
	*Grade 2*	10–50 neutrophils
	*Grade 3*	>50 neutrophils
